# Chronicling the June *conundrum*

**DOI:** 10.1007/s10194-011-0365-x

**Published:** 2011-07-15

**Authors:** Paolo Martelletti

**Affiliations:** Department of Medical and Molecular Sciences, Sapienza University of Rome, Rome, Italy

## Rethinking JHP’s evolution

The publication on June 28 2011 of the Journal Citation Reports^®^, with the new Impact Factor (IF) 2010, based on the citations that every journal indexed in Sci-Search has received in the previous biennium, has shown how the arrival of The Journal of Headache and Pain (JHP) in the headache scientific arena has not been a meteor. Hence some hasty catastrophic previsions have been neither correct nor reasonable. The stabilization of the IF 2010 on the value of 2.015 has reconfirmed that the reliance in the contents of the journal is constant and well placed. On behalf of the whole great team of people who work at this project, I express the sense of honor and the great satisfaction for the service rendered to the scientific community.

## Stabilization phase of the IF value

After the JHP appearance, just 2 years ago, in the international IF ranking and the following push towards a rise, a phase of stabilization and consolidation of the results is welcomed and reassuring [1, 2]. The analysis of the contents that have brought to today’s evaluation of the IF is food for thoughts (full analytical data are available as ESM):

(i) The number of citations has increased by 20% as compared to 2009
(ii) The number of accepted papers has increased to 70% as compared to 2009
(iii) The number of submitted papers has increased to 71% as compared to 2009
(iv) The rejection rate has remained steady at the value of 55%

Taking into account that we have fully recognized an increased quantity (quality) of the submitted papers (iii) and it has resulted in a higher number of accepted papers (ii), making vain the point relative to the citations (i). This, however, witnessed the higher interest that the readership keeps showing towards JHP.

## SpringerOpen fall-out

The much debated and feared opening towards the strategy of open access to contents for everyone, without costs, has been feasible through the utilization of various grants. I thank European Headache Federation, Lifting The Burden, Sapienza University, Springer and advertisers, who made this choice possible. Consequently, the visibility of JHP has had a sudden and violent rise. In fact, in the first 4 months of this new SpringerOpen model, the rate of downloaded papers has risen up by 60%. All this has accomplished a much wider educational mission of every paper and therefore of JHP also, and lays strong basis for a positive fall-out in terms of number of citations on the next evaluation in 2012.

## Future strategies

It is now important to attract more and more high-value reviews, original papers, basic science papers or tutorials. These are the pillars of the editorial development of JHP. There is a need to attract also updated therapeutic guidelines, new classification proposals, or position papers, or RCTs, which are proved to be a great boost factor for the IF indexes. But these are field choices for the affiliated societies.

Anyways, from 2012 JHP will appear with eight issues per year and the articles, now freely available online from the SpringerLink platform [3], will additionally be accessible from the PubMed Central Journal List [4]. The latter is a free full-text archive of biomedical and life sciences journal literature at the U.S. National Institutes of Health’s National Library of Medicine (NIH/NLM). This will represent a further extraordinary access point, allowing a much wider diffusion of JHP contents.

Every year the *conundrum* of IF fills the month of June with hopes, doubts and expectations. JHP does not live this event with less anxiety; the Journal, beyond its possible imperfections, attributes real values to the work and the choices of all of us: Editors, Board Members, Referees, Researchers, Readers, Publisher.

I think this is the staging post for every scientific journal, to serve the civil society helping it to reach its scientific, educational and health care unmet needs.

## Electronic supplementary material

Below is the link to the electronic supplementary material.

Supplementary material (PDF 52 kb)
