# Supplementary material for: Chronicling the June conundrum
Source: J Headache Pain. 2011 Jul 15;12(4):397–8. doi: 10.1007/s10194-011-0365-x (PMC3139055; doi:10.1007/s10194-011-0365-x)
Supplement: Supplementary file 1 — Supplementary material (PDF 52 kb) [file 10194_2011_365_MOESM1_ESM.pdf]

Electronic Supplementary Material

The Journal of Headache and Pain - 2010				
IF	Immediacy Index	Cited Half-life	Total Cites	Eigenfactor™ Metrics
2.015	0.603	3.2	661	0.00284
**Subject Category *Neurosciences***				
Median IF	Aggregate Immediacy Index	Aggregated Cited-Half-Life	JHP Ranking	Quartile
2.783	0.724	7.2	161/237	Q3
**Subject Category *Clinical Neurology***				
Median IF	Aggregate Immediacy Index	Aggregated Cited-Half-Life	JHP Ranking	Quartile
1.994	0.535	7.0	91/185	Q2
**ΔIF JHP vs Median IF Neurosciences = -0.768 and vs Median IF Clinical Neurology = +0.021**				
Source: Journal Citation Reports (2010 JCR Science Edition) http://admin-apps.isiknowledge.com/JCR/JCR<br>Accessed, June 30th, 2011				
